# Pulmonary Tuberculosis and Lepromatous Leprosy Coinfection

**DOI:** 10.1155/2015/898410

**Published:** 2015-10-04

**Authors:** F. A. Sendrasoa, I. M. Ranaivo, O. Raharolahy, M. Andrianarison, L. S. Ramarozatovo, F. Rapelanoro Rabenja

**Affiliations:** Department of Dermatology, Joseph Raseta Befelatanana Hospital, 101 Antananarivo, Madagascar

## Abstract

Simultaneous occurrence of leprosy and pulmonary tuberculosis is reported infrequently in the modern era. We report a case of pulmonary tuberculosis diagnosed in patient being treated with glucocorticoids for complications of leprosy (type II reaction). Physicians should recognize that the leprosy patients treated with glucocorticoid may develop tuberculosis.

## 1. Introduction

Leprosy and tuberculosis are two pathogens, which have been identified as infecting humans 9 000 and 4 000 years ago, respectively. They remain endemic in Madagascar, and the annual new case detection rates of leprosy and tuberculosis were 8 per 100 000 and 233 per 100 000, respectively. So, 2–6 cases of concomitant infection per 100 000 populations should be detected, in one year. However, no report of concomitant infection was identified in Madagascar. We aim to report a case of 49-year-old man who presented with pulmonary tuberculosis and lepromatous leprosy coinfection.

## 2. Case Presentation

**Figure 1:**
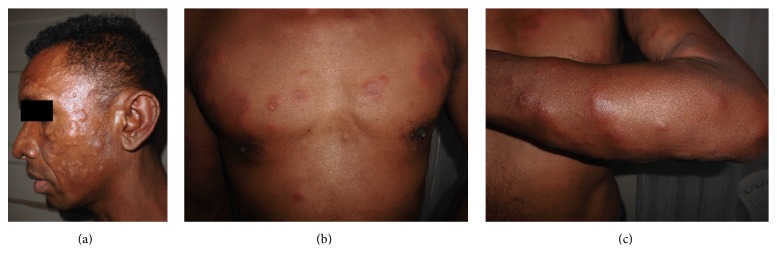
(a, b) Nodular lesions over face and trunk. (c) Nodular lesions over forearm.

**Figure 2:**
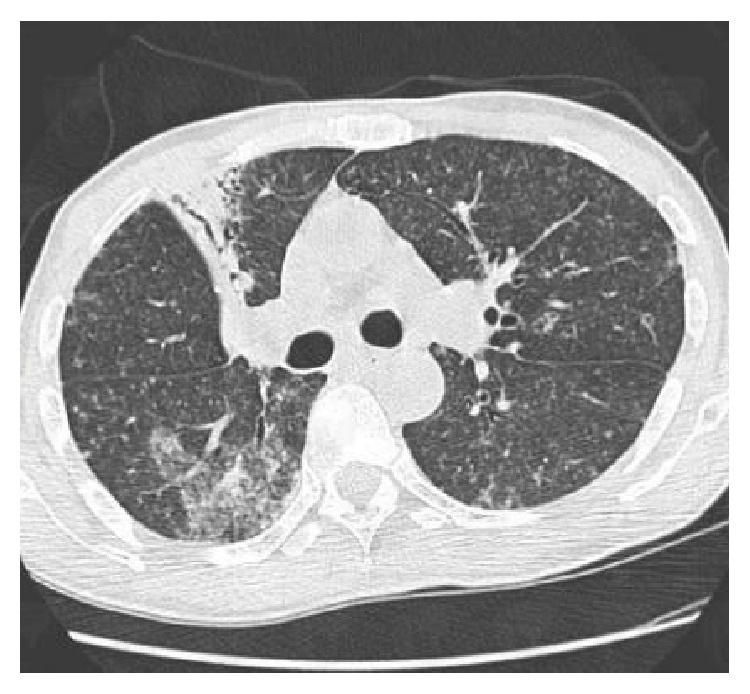
Alveolar-interstitial opacities at the left lower lobe.

A 49-year-old man, nonsmoker, was admitted to dermatology department and followed up for diffuse lepromatous leprosy. He was vaccinated with BCG and he had no past history of tuberculosis. Diagnosis of leprosy was documented based on histological and bacteriologic evidence: a slit skin smear from the ear lobe was positive for lepra bacilli (BI3+), histopathology from the lesion on the face showed granulomas consisting of epithelioid histiocytes and lymphocytes with central caseous surrounding vessels and nerves, and PCR of biopsy specimens were positive for* Mycobacterium leprae*. After successful treatment using dapsone (100 mg/day), rifampicin (600 mg/month), and clofazimine (300 mg/month and 50 mg/day) during twelve months, hypopigmented skin lesions on the trunk and congestive rhinitis disappeared and the slit skin smear was negative. One month after the end of the treatment, he presented with diffuse papulonodular lesions on the face and trunk, fever, and alteration of general status. On the basis of his symptoms, diagnosis of leprosy reaction (type II) was made and the patient was treated using prednisone at a dose of 40 mg/kg/day. Outcome was unfavorable after two months of corticotherapy, and we had to wait for two supplementary months before we could get clofazimine to add corticoid. After 1 month of this treatment, he presented with fever, weight loss, and asthenia. Clinical examination revealed fever of 39°C, nodular lesions over face, trunk, forearms, and dorsum of hands (Figures 1(a), 1(b), and 1(c)). He presented no neurologic impairment. Biological examination showed inflammatory syndrome: CRP 393 mg/L, total leukocyte count 16,2 × 10^9^/L, neutrophilia 15,3 × 10^9^/L, and lymphopenia 0,48 × 10^9^/L. Serum creatinine and alanine aminotransferase were normal. HIV status was negative. Two out of three sputum samples were positive for acid fast bacilli. Chest tomography showed alveolar-interstitial opacities at the left lower lobe (Figure 2). Bronchoscopy detected thickening of lower lobar bronchi, without malignancy in histopathology of biopsy specimens. The patient was treated by antitubercular treatment. One month after the onset of this treatment, there were only two nodular lesions on the trunk, fever disappeared, and general status improved.

## 3. Discussion

Concomitant pulmonary tuberculosis and leprosy case is uncommon, even in countries like Madagascar where both mycobacterial infections are endemic. On review of data from three leprosy referral centres in Hyderabad, India, from 2000 to 2013, three cases of this coinfection were identified [1]. To our knowledge, there have been no reported cases of concomitant pulmonary tuberculosis and leprosy in Madagascar.

Kumar et al. studied 117 patients of leprosy for evidence of concomitant tuberculosis. Nine patients (7,7%) showed evidence of active tuberculosis, bacteriologically and radiologically. Tuberculosis was found to occur throughout leprosy spectrum [2]. The interaction between leprosy and tuberculosis and their repercussions on the incidence of each other still remain a matter of debate [3].

The diagnosis of pulmonary tuberculosis was clinicoradiological and bacteriological in our patient. Mantoux test was not available because only one center had Mantoux test in Madagascar and it is very expensive.

The gap duration between the development of leprosy and tuberculosis ranged from 2 months to 10–15 years, and the study with largest data showed gap duration of about 10–15 years, where duration of tuberculosis in most of the cases was within six months (while in present case it was 17 months). Only two cases of tuberculosis were found to occur earlier than leprosy [4], as one study concluded that tuberculosis can occur during full spectrum of leprosy.

In case of leprosy, corticosteroids are used primarily in the treatment of type I and type II reactions and silent neuropathy. Rawson et al. reported development of pulmonary tuberculosis after corticosteroid intake in two cases of leprosy [1]. Prasad et al. reported also concomitant pulmonary tuberculosis and borderline leprosy with type II lepra reaction in a single patient who received corticosteroid for more than 3 months [5]. However, major trials of steroid treatment in multidrug therapy for leprosy, such as the TRIPOD studies, have failed to identify development of tuberculosis in some 300 patients who were followed up for over 24 months [6, 7]. This result may be correlated with low doses of prednisolone (around 20 mg/day). Dosing used in our case can be greater than this even if the duration of treatment was not long. Literature defined that steroid was for a minimum of 16 weeks to treat leprosy reactions.

Table 1 shows some cases of concomitant tuberculosis and leprosy reported in the literature.

## 4. Conclusion

Our patient's case illustrates an uncommon occurrence of concomitant pulmonary tuberculosis and leprosy, presumably the first reported case in Madagascar, and shows the increased risk of pulmonary tuberculosis in patients with leprosy treated with glucocorticoids. Therefore, it becomes imperative for physicians treating leprosy complications with steroids to have a high degree of suspicion to diagnose pulmonary tuberculosis.

## Figures and Tables

**Table 1:** Comparative analysis of some cases of leprosy-tuberculosis coinfection reported by various authors for last ten years.

	Sreeramareddy et al. [8]		Prasad et al. [5]	Trindade et al. [9]		Present author
Number of cases	2		1	2		1
	Case I	Case II		Case I	Case II	
Age	65	50	34	31	46	49
Gap duration between leprosy and tuberculosis	3 M	2 Y	11 M	6 M	1 M	17 M
Types of leprosy	BL	LL	BL	BB-BT	BT-BB	LL
First infection	Leprosy	Leprosy	Leprosy	Tuberculosis	Leprosy	Leprosy
Past history of tuberculosis	No	NA	NA	NA	NA	No
Risk factors	Corticosteroids	Corticosteroids	Corticosteroids	NA	Corticosteroids	Corticosteroids
Types of tuberculosis	Pulmonary	Pulmonary	Pulmonary	Pleural TB	Pulmonary	Pulmonary
Chest radiographs // chest tomography	Pleural effusion + bilateral infiltrates	Cavitary lesion + bilateral infiltrates	Cavitation + fibroconsolidation	Pleural effusion	Parenchymal opacification	Alveolar-interstitial syndrome
Sputum	Positive	Positive	Positive	NA	Positive	Positive
Diagnosis of leprosy	NA	NA	Slit skin smear	Histopathology + Fite-Faraco	Histopathology	Histopathology
Lepra reaction	No	Type II	Type II	Type I	Type I	Type II

M: month; Y: year; NA: data not available; lepra reaction type I (reversal); lepra reaction type II (ENL).
